# Rating Player Actions in Soccer

**DOI:** 10.3389/fspor.2021.682986

**Published:** 2021-07-15

**Authors:** Uwe Dick, Maryam Tavakol, Ulf Brefeld

**Affiliations:** ^1^Machine Learning Group, Leuphana University of Lüneburg, Lüneburg, Germany; ^2^UAI Group, Eindhoven University of Technology, Eindhoven, Netherlands

**Keywords:** sports analytics, soccer, graph networks, trajectory prediction, trajectory data

## Abstract

We present a data-driven model that rates actions of the player in soccer with respect to their contribution to ball possession phases. This study approach consists of two interconnected parts: (i) a trajectory prediction model that is learned from real tracking data and predicts movements of players and (ii) a prediction model for the outcome of a ball possession phase. Interactions between players and a ball are captured by a graph recurrent neural network (GRNN) and we show empirically that the network reliably predicts both, player trajectories as well as outcomes of ball possession phases. We derive a set of aggregated performance indicators to compare players with respect to. to their contribution to the success of their team.

## 1. Introduction

Analyzing team and player behavior in soccer games is an inherently difficult task: coordination among teammates is highly complex and even well-developed game plans can be negated by a single bad pass. In these circumstances, the contribution of a single player to the success of a team is often difficult to estimate. While obvious decisive actions, such as scoring and assisting a goal, losing the important tackle just before the scorer takes the shot, can easily be measured and attributed to those players^1^, other aspects are often left for evaluation by experts and coaches. For example, does a player give too much space to the attacking midfielder in certain situations, or does a striker regularly wait too long after losing the ball before they track back and try to slow down the attack of the opponent?

Last but not least, there is a great deal of randomness in the game of soccer and the outcome highly depends on chance. A good team may still lose the game simply because they hit the post instead of the goal, while their opponents capitalize on an individual error. Still, the good team may have created more scoring opportunities and played better in general and when it comes to player rating, the players of the good team should be rated accordingly.

Because of the complexity in which a soccer match develops, there has been a lot of research on specific aspects of the game to analyze and measure their potential influence on the outcome. For example, Spearman et al. (2017) developed a model for pass probabilities, Fernandez and Bornn (2018) measured the space generated by players, Spearman (2018) and Link et al. (2016) analyzed scoring opportunities, Dick and Brefeld (2019) learned general values of game situations, and Fernández et al. (2019) analyzed the value of certain areas of the pitch depending on the game situation. Another aspect that received attention is learning player movements from trajectory data. Brefeld et al. (2019) learned general distributions of player movements without taking into account any context including positions of other players or the ball, whereas Le et al. (2017b) and Yeh et al. (2019) aimed at simulating player trajectories. While the former can be used to compute zones of control of players, i.e., the zones that are defined as the area that a player will most likely occupy at a certain time in the future, the latter can be used to perform counterfactual analysis to compute how a player could have influenced a situation if they had moved differently (Le et al., 2017a).

In this article, we devise a model that predicts player trajectories and estimates the expected outcome of a ball possession phase. A ball possession phase is a time between a team gaining possession of the ball and loosing it again. Ultimately, the outcome of such a sequence could either be a success by scoring a goal or a failure by losing possession to the opponent. However, as goals are rather rare in soccer, success can also be defined less precisely, e.g., by the expected goal value in case of a shot or, even more general, by entering a certain area on the pitch that potentially allows for a scoring chance. In this article, we use the latter approach and consider a built-up play, a success if the team enters a dangerous zone while retaining ball possession. The basis for the model is a graph recurrent neural net (GRNN) that models interactions between players and the ball using a fully-connected graph, where all players and ball are represented by nodes. Their interdependencies are given by edges between the nodes and are, finally, learned by the GRNN to predeict the outcome of a ball possession phase. To this extent, the model also learns node features for every node to predict the movement of the corresponding player or ball. This research borrows ideas from Dick and Brefeld (2019) to learn developing values from game situations but we show that using a graph model significantly improves the ability to predict successful outcomes. It also extends the study of Yeh et al. (2019) who use a graph recurrent network to predict trajectories of soccer players. In contrast with their models, we use a different way of realizing multimodal movement distributions.

We learn and evaluate the model on data from 54 Bundesliga matches where ball and player positions are captured at 25 frames per second^2^. Those data were augmented by event data containing manually annotated information about passes, shots, tackles, etc. Together, tracking and event data constitute official match data and are provided by Deutsche Fuball Liga (DFL) ^3^. We show empirically that the predictions of success of this approach mirrors the likelihood of success during a ball possession pretty well, both quantitatively and qualitatively. Furthermore, this model can predict player trajectories that can be turned into an “average” Bundesliga player. We derive a set of performance indicators that can be used to assess the performance of individual players' objectively while going beyond simple metrics, such as goals or assists. We also show that success predictions can be used to query match databases for important game segments where a single action, such as a pass or a dribble, significantly changes the likelihood of a successful attack, i.e., it allows for extracting potentially game-changing individual plays automatically.

The remainder of the article is structured as follows. We briefly review related work in the next section and present the main contribution in section 3 on empirical results in section 4, and section 5 provides a discussion of practical applications in section 5, and give conclusion in section 6.

## 2. Related Work

Rating game states and player actions in soccer is a fairly new topic that aims at quantitatively measuring how particular actions, such as passes or dribbles, influence the outcome of a game or how likely a game configuration consisting of ball and player positions, control of the ball, or recent actions, among others, may lead to a scoring opportunity. Data sources used to ground models on observable data generally consist of event data, such as information about shots and passes and/or trajectory data that measures the position of players and the ball several times a second. Due to the inherent complexity of soccer matches, most work has focused on rather specific aspects of soccer to rate those particular actions, such as passes or shots. Shot opportunities were investigated using the expected goal (sometimes also called xGoal or xG) value, e.g., in Lucey et al. (2014) with the control of a team ON that point and in Spearman (2018) who developed a model that estimates off-ball scoring opportunities by combining scoring probabilities from a certain point on the pitch with the control of a team that point and the probability that the ball will reach the point. Link et al. (2016) developed a model that estimates the dangerness of a game state by a combination of position, pressure, control, and density of future positions. Work on the analysis of passes in soccer included study by Power et al. (2017), who compared the risk of a pass (probability of an intercepted pass) vs. its reward, the likelihood that the attacking team will take a shot at goal within 10 s after the pass. Goes et al. (2019) estimated the effectiveness of passes by measuring how many defensive players have to move and how much their defensive organization reduces following a pass. Spearman et al. (2017) developed a model that estimates the probability that a pass to a teammate is successful. Other related work consists of defining game indexes (McHale et al., 2012), measuring the space occupation and generation by players and teams (Fernandez and Bornn, 2018), and deploying frequency-related criteria to narrow down the candidate space of interesting patterns (Haase and Brefeld, 2014).

More general approaches include a study by Fernández et al. (2019) who developed an expected possession value that describes the likelihood that a ball possession ends in a team scoring a goal. The value is decomposed into three potential actions: passes, shots, and ball-drives. Models that estimate the expected success after performing the actions are learned for each action individually, though no further specifics of those models were presented. The work is more focused on the application of the model rather than technical detail, whereas this current work instead focuses on presenting a concise method for computing the value at any point in time during a soccer match, thereby allowing to rate any action based on the difference between values before and after it is performed. In that way, it is more closely related to Decroos et al. (2019) who learned values for actions, such as passes and dribbles, based on event data. The probability of scoring a goal after a certain action is performed is estimated by learning a binary classifier on a set of handcrafted features of actions. This work is similar in spirit to the model presented in the current work but only uses event data instead of trajectory data. Instead, the approach on learning to rate states and actions is partly based on Dick and Brefeld (2019), who used a convolutional neural network to learn ratings of game situations using ideas from reinforcement learning.

Another aspect of soccer analytics that received attention is the simulation of player trajectories. Peralta Alguacil et al. (2020) partly combine rating player actions and movement prediction by identifying potential runs or actions of players that maximize a combination of three factors. Players may aim at running into a position that maximizes the probability of receiving a pass, based on the work of Spearman et al. (2017). Players may run into a position that maximizes pitch impact, a proprietary measure for how dangerous a position is, or they may want to maximize the pitch control measure of Fernandez and Bornn (2018), the amount of space controlled by a team. They use the physics-based approach of Fujimura and Sugihara (2005) to estimate which positions a player may reach in time for a pass and sample from inside this area find optimal positions. This approach does not have the aim to simulate real player behavior but instead to compute alternative movements that can be presented to players and coaches analyze collective behavior. Another way to model general player movements is to use a counting-based approach to estimate a probabilistic movement model that predicts the density of future positions for short-time windows (Brefeld et al., 2019). However, this approach cannot be used to simulate player trajectories because it does not take into account any context of other players or the ball.

Several studies instead investigated learning to predict player trajectories in soccer and other sports, such as basketball (Le et al., 2017a,b; Yeh et al., 2019; Zhan et al., 2019). A general problem when learning coordinated movements of several agents, such as players in a team, is that observed trajectories come as unordered sets of individuals. When learning from a variety of games, there is a need to incorporate different teams and players and, consequently, the model has to work without a natural ordering of the players. Le et al. (2017b,a) learned trajectories of players by estimating their roles in a given episode and then use these role assignments to predict future movements given the role. A similar approach has been taken by Zhan et al. (2018, 2019) who also used role assignments to predict future positions. Their results for basketball players are computed using a variational recurrent neural network (RNN) that allows for the inclusion of macro goals. Similarly, Felsen et al. (2018) computed tree-based role assignment to predict trajectories using a conditional variational autoencoder.

In general, graph representations suggest themselves to model interactions of players and ball. In one way or another, players and balls are identified with nodes in a fully connected graph, where edge weights correspond to their interaction and are learned in the training process. For example, Yeh et al. (2019) proposed to leverage graph neural networks (GNN), which are naturally suited to model coordinated behavior because of their invariance to permutations in the input. The authors proposed a graph variational RNN to predict future positions of soccer and basketball players and showed that graph networks outperform other models when predicting player trajectories. Similarly, Hoshen (2017) and Kipf et al. (2018) proposed graph-related attention mechanisms to learn Basketball player trajectories.

Graph neural networks have been widely used to model structured or relational data, refer to Battaglia et al. (2018) for an overview. In cases where data is sequential in nature, GRNN have been widely deployed, for an example, to mix graph representations with recurrent layers (Sanchez-Gonzalez et al., 2018; Yeh et al., 2019), such as gaited recurrent units (GRU, Cho et al., 2014).

Due to the complex nature of movements in soccer, a natural assumption on the distribution of future positions is its multi-modality. Trivially, any probabilistic model that aims to predict future positions in team sports needs to reflect the multi-modal nature in some sense. Hence, Zhan et al. (2019), Zhan et al. (2018), Yeh et al. (2019), and Felsen et al. (2018) use conditional variational models (CVM) with Gaussian emission functions to account for multi-modality in the data. However, Graves (2013) has shown that learning a Gaussian mixture model (GMM) as output distribution, by combining recurrent neural networks (RNNs) with mixture density networks (MDNs) Bishop (1994), yields good results for spatiotemporal tasks. Indeed, Rudolph et al. (2020) has recently shown that combining GMM emissions with recurrent graph networks performs at least on par with more complex CVM In this article, the approach for learning soccer player trajectories is based on the study of Yeh et al. (2019) (s.a.) in that we use a GRNN model with typed edges but instead of a CVM approach we use an MDN output to model multi-modality. That way, we make use of the findings of Rudolph et al. (2020) and also of Yeh et al. (2019) who in their experiments found out that variational models do not outperform non-variational models on soccer data.

## 3. Learning to Predict and Rate Actions

At each point in time during a soccer match, all players and the ball interact with each other. For example, a defender reacts to a run of the closest opponent, the player in ball possession passes the ball to a teammate, another player moves away from an opponent to be available for receiving a pass, a defender positions oneself to minimize the likelihood that a potential counterattack would lead to a dangerous situation. Hence, each player decides on their next action, a run with or without the ball, a tackle, or a potential on-ball action, based on previous and current actions of other players.

At the same time, actions of the palyer are aimed at maximizing the likelihood of winning the game which, at a specific point in time, could be best achieved by either a tackle, a run into open space, a pass, a shot, or even by committing a foul. It is, therefore, often beneficial to split a soccer game into separate phases based on which team has possession of the ball. Ultimately, such a ball possession phase ends when the team either looses possession of the ball, when the ball is out of play, or when a team scores a goal, thereby also loosing possession of the ball. That way, the likelihood of winning the game can be split into separate phases with likelihoods of scoring in each phase. Indeed, at each point in time during a ball possession phase, there is a likelihood of scoring without losing the ball before. Each player of the ball possessing team aims at increasing this likelihood with their actions, whereas the players of the defending team aim at decreasing it.

In this article, we focus only on the movement actions of players with or without the ball and hence predict player trajectories based on the trajectories of other players and the ball. Regarding success prediction, goals are scarce in soccer which causes a general problem of predictability of soccer match outcomes. For example, during the 2017/18 Bundesliga season, teams took on average 27 shots per game but only 2.79 were converted into goals. We use data from 54 Bundesliga games from that season 2017/18, with a total of 148 goals and 1450 shots (i.e., 1 goal out of 9.8 shots). Several of those shots and goals originated from set pieces and are not suitable for use as an outcome of a ball possession phase. As a consequence, we focus on a proxy that appears more frequently in the data to facilitate learning with more evenly balanced success signals. Thus, instead of defining success simply by goals or shots, we propose to consider ball possession phases “successful” that ends with the ball possessing team entering a *dangerous zone*. In the remainder, we use the area that spans until 2 m outside the penalty box as the dangerous zone. This signal appears 1,879 times in the data and serves a good proxy for success.

We will learn player actions and success prediction separately but use the same underlying model for both so that they can be used together to rate actions. The common basis for the model is a GRNN. Graph networks have shown excellent results in multi-agent prediction problems, e.g., for predicting movements of soccer and basketball players (Yeh et al., 2019). The main idea is to represent all *agents*, that is players and the ball, as nodes in a (fully connected) graph. Interactions and interdependencies between these agents are encoded by edges between pairs of agents. In addition, we include a graph state of the graph which encodes information about the whole graph, cmp. e.g., Battaglia et al. (2018). The output of a graph network is a set of node features that encode information about each agent and the graph state features.

Overall, the model operates as follows: information about players and the ball, such as position, velocity, and team association, are fed into a GRNN which computes node and graph state features. Node features associated with a particular player are used to predict their movement. This prediction of the next action is computed simultaneously for all players. Graph state features are used to predict whether the current ball possession leads to a success. The GRNN is described in detail in the next subsection, and section 3.2 presents how movement and success predictions are learned from observed data.

Let the state of player *i* at time index *T* be denoted by $S_{T}^{i}$. The state $S_{T}^{i}$ contains position of the player $x^{i}\in R^{2}$ in *xy*-coordinates, velocity of the player, and indicators whether the team of the player is in possession of the ball or the player itself has currently ball possession. In addition, the state encodes whether the team of the player plays in positive *x*-direction or vice versa or the player is a goalkeeper. Superscript 0 is reserved to index the state of the ball $S_{T}^{0}$ with its position and velocity at time *T*. We sometimes aggregate states of all players and ball at time *T*, denoted by $S_{T}^{0:N}$, where usually *N* = 22, as well as consecutive state sequences from the beginning of a sequence until *T* by $S_{:T}^{i}$.

### 3.1. Graph Model

Graph models encode information about all players and the ball using a fully connected graph representation. Each player or ball *i* is represented by a node *v*^*i*^ and nodes *i* and *j* are connected by directed edges *e*^*ij*^. In addition, state features *u* represent information about the whole graph.

**Figure 1:**
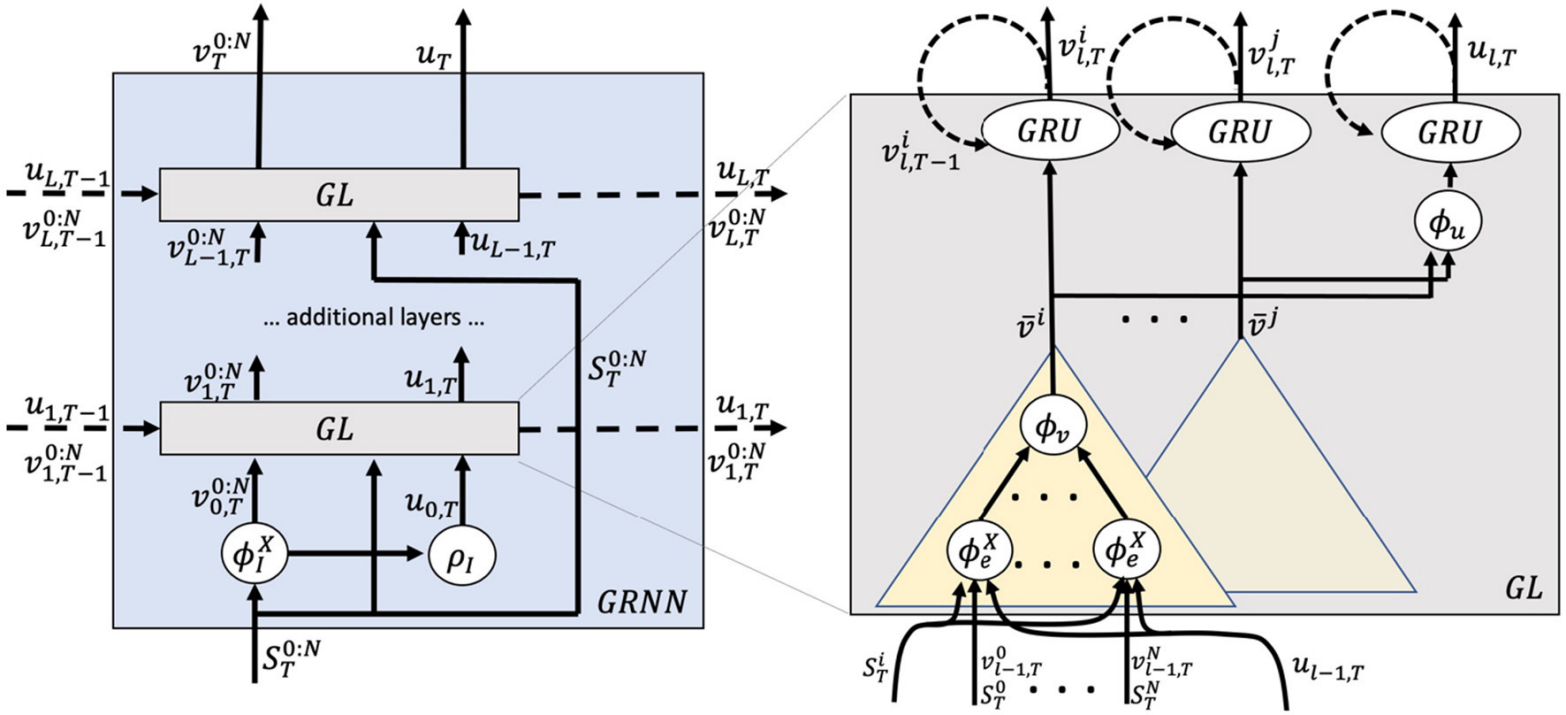
Graphical representation of the graph recurrent neural net (GRNN).

This GRNN consists of several graph layers (GL) that each encodes the whole graph and transforms node and state features based on information of the previous time step and input features. Figure 1 shows a graphical representation of the GRNN and GL. The input to the GRNN is a sequence of player and ball states $S_{:T}^{0:N}$, and the outputs consist of sequences of node features $v_{:T}^{0:N}$, where each $v_{T}^{i}$ denotes features of player *i* at time *T*. In addition, the GRNN outputs a sequence of graph state features *u*_:*T*_.

The big picture is as follows: At each time step, input features $S_{T}^{0:N}$ are fed into initial feature functions ϕ_*I*_ and ρ_*I*_ to create initial node and graph state representations $v_{0,T}^{0:N}$ and *u*_0, *T*_. Those initial features are passed through multiple recurrent GL to output final node features $v_{T}^{0:N}$ and graph state features *u*_*T*_. To provide more details, we begin by presenting the basic steps that are performed in a single GL ℓ.

At first, all edge features $e_{\ell ,T}^{ij}$ are computed using an attention mechanism from the corresponding node $v_{\ell -1,T}^{j}$ and state features *u*_ℓ−1, *T*_ from the previous layer ℓ−1 and input features $S_{T}^{i},S_{T}^{j}$. Edges are assigned a type according to whether they connect player to player, player to ball, or ball to player, as proposed in Yeh et al. (2019). Each edge type has its own set of parameters. The edges are aggregated into intermediate node features ${\bar{v}}_{\ell ,T}^{i}$ which are fed into standard (GRU, Cho et al., 2014) to produce transformed node features $v_{\ell ,T}^{i}$. In addition, intermediate node features of all nodes are aggregated into intermediate state features ū_ℓ, *T*_ using another attention mechanism which is also fed into a GRU unit to compute transformed state features *u*_ℓ, *T*_. Equations (1) to (5) show the detailed computations to compute transformations $\left(v_{\ell ,T}^{0:N},u_{\ell ,T}\right)=GL\left(v_{\ell -1,T}^{0:N},u_{\ell -1,T},v_{\ell ,T-1}^{0:N},u_{\ell ,T-1},S_{T}^{0:N}\right)$.

(1) $$\begin{array}{l} e_{\ell ,T}^{ij}=\phi_{e}^{type}\left(S_{T}^{i},S_{T}^{j},v_{\ell -1,T}^{j};\theta_{\ell }\right)\\ \;=\alpha_{\ell }^{type}\left(\left[S_{T}^{i}-S_{T}^{j},u_{\ell -1,T}\right]\right)⊙v_{\ell -1,T}^{j} \end{array}$$

(2) $$\begin{array}{r} {\bar{v}}_{\ell ,T}^{i}=\phi_{v}\left(\left\{e_{\ell ,T}^{ij},j=0,...,N\right\}\right)=f_{v}\left(\overset{N}{\underset{j=0}{\sum }}e_{\ell ,T}^{ij}\right) \end{array}$$

(3) $$\begin{array}{r} {ū}_{\ell ,T}=\phi_{u}\left(\left\{{\bar{v}}_{\ell ,T}^{i},j=0,...,N\right\},S^{0:N}\right)=\overset{N}{\underset{i=0}{\sum }}\alpha_{\ell }^{v}\left(S_{T}^{i}\right)⊙{\bar{v}}_{\ell ,T}^{i} \end{array}$$

(4) $$\begin{array}{r} v_{\ell ,T}^{i}=GRU\left({\bar{v}}_{\ell ,T}^{i},v_{\ell ,T-1}^{i}\right) \end{array}$$

(5) $$\begin{array}{r} u_{\ell ,T}=GRU\left({ū}_{\ell ,T},u_{\ell ,T-1}\right) \end{array}$$

Attention functions, α^*type*^ and α^*v*^, and function, *f*_*v*_, are implemented with multilayer perceptrons (MLP) with ReLU activations and ⊙ denotes the element-wise product. The initial node and state features, $v_{0,T}^{i}$ and *u*_0, *T*_, that serve as input to the first GL are computed by Equations (6) and (7) from the input features,

(6) $$\begin{array}{r} v_{0,T}^{i}=\phi_{I}\left(S_{T}^{i}\right) \end{array}$$

(7) $$\begin{array}{r} u_{0,T}=\rho_{I}\left(\left\{v_{0,T}^{i},j=0,...,N\right\}\right)=\overset{N}{\underset{i=0}{\sum }}v_{0,T}^{i} \end{array}$$

where feature function ϕ_*I*_ is realized by a two-layer MLP. Overall, the GRNN transforms input features to node and graph state features, i.e., $\left(v_{:T}^{0:N},u_{:T}\right)=GRNN\left(S_{:T}^{0:N}\right)$.

### 3.2. Prediction

**Movements:** The prediction of the movement of player *i* at time *T* is based on node features $v_{T}^{i}$. In accordance with Rudolph et al. (2020), the movement distribution is given by an MDN to predict the relative movement Δ*x* of *i* in time *t*, that is $\Delta x_{T}^{i}=x_{T+t}^{i}-x_{T}^{i}$, where $x_{T}^{i}$ is the *xy*-position of player *i* at time *T*. MDNs model the output distribution with a GMM with *K* mixtures is given by the Equation (8)

(8) $$\begin{array}{r} p^{i}\left(\Delta x|t,S_{T}^{i}\right)=\overset{K}{\underset{k=1}{\sum }}\pi \left(k|t,v_{T}^{i}\right)N\left(\Delta x|\mu_{k}\left(t,v_{T}^{i}\right),\sigma_{k}\left(t,v_{T}^{i}\right)\cdot I\right) \end{array}$$

The categorical mixture distribution π is modeled using a two-layer MLP with ReLU activations and a standard softmax output. Similarly, Gaussian means $\mu_{k}\left(t,S^{i}\right)\in R^{2}$ and variances $\sigma_{k}\left(t,S^{i}\right)\in R^{2}$ are predicted using two-layer MLPs with ReLU activations and linear and exponential output activation functions, respectively, where *I* is the identity matrix. The model is learned by minimizing the log-likelihood of observed relative movements.

**Success:** Predicting the success during a ball possession phase demands information about all contributing parties and the natural choice is to use the graph state *u*_*T*_ to estimate whether the attacking team can successfully conclude the phase. The approach is to use ideas from reinforcement learning and learn a value function (V) that maps states to the expected (discounted) reward of a possession phase. The reward $R\left(S_{T}^{0:N}\right)\in \left\{-1,0,+1\right\}$ in state $S_{T}^{0:N}$ is defined as +1 if the team playing in positive *x*-direction completes their ball possession, i.e., the ball $S_{T}^{0}$ is in a position inside the dangerous area and the team is in ball possession; and −1, if the team playing in the other direction completes successfully; and 0, otherwise. A *V* is defined as the expected discounted future reward from state $S_{T}^{0:N}$, that is, $V\left(S_{T}^{0:N}\right)=E_{S_{T:T_{E}}^{0:N}}\left[\overset{T_{E}-T}{\underset{t=0}{\sum }}\gamma^{t}R\left(S_{T+t}^{0:N}\right)\right]$. The expectation is taken over all possible futures how the ball possession may unfold from time *T* until it ends at some time *T*_*E*_ and γ∈(0, 1) is a discount factor. The effective V range thereby is between –1 and 1. We model the V using the graph state as $V\left(S_{T}^{0:N}\right)=\phi_{V}\left(u_{T}\right)$, where ϕ_*V*_ is a two-layer MLP with ReLU activations and linear output. The model is learned with the lambda-return algorithm (Sutton and Barto, 1998; Dick and Brefeld, 2019) on observed ball possession phases.

## 4. Empirical Results

We evaluate the performance of the model on trajectory data from 54 games of the 2017/18 season of men's Bundesliga. For each game, we have access to the sequence of *x* and *y*-coordinates of all players and the ball sampled at 25 frames per second. We approximate the speed and direction of players and ball at each frame by computing the differences in positions over the last 0.12 s. In addition, we have access to event data that includes passes, tackles, etc.

We extract all episodes of open play where one team continuously retains ball possession without the game being halted. Each such ball possession phase ends with the team controlling the ball either losing the ball or the play is stopped, or with that team performing a “*success action*.” An episode is considered successful if the team carries the ball into an area that extends 2 m outside the penalty box of the opponent. In case of a successful action, the episode is labeled with a positive reward of 1 if the team in possession of the ball plays from left to right (in the positive x-direction), a reward of -1 if the team plays from right to left, and a zero reward otherwise. Overall, the data consists of 10,626 ball possession phases with an average length of 10.6 s 1,879 of those possession phases have a non-zero success signal. We report averages of 5-fold cross-validations; error bars indicate SE.

The architecture of the GRNN used in the experiments consist of two *GL*, where both layers have a GRU width of 1, 024 and the attention function, $\alpha_{l}^{type}$, $\alpha_{l}^{v}$, as well as function, *f*_*v*_, possess a single hidden layer with 512 units. Input feature function ϕ_*I*_ has two layers with 128 and 256 units, respectively. The MDN consists of a Gaussian mixture with *k* = 6 components and GMM functions, π, μ_*k*_, and σ_*k*_, each have two layers with 512 units. The same holds for ϕ_*V*_. ^4^

We focus on the evaluation on three aspects: section 4.1 verifies whether the learned V translate to actual dangerous situations; section 4.2 evaluates the quality of the predicted player trajectories; and section 4.3 shows how both aspects can be combined to allow the assessment of player performance.

### 4.1. Value Function and Success

The V assigns values to situations on the pitch. The higher the absolute value of the V, the more likely a team should be able to successfully finish a ball possession phase.

**Figure 2:**
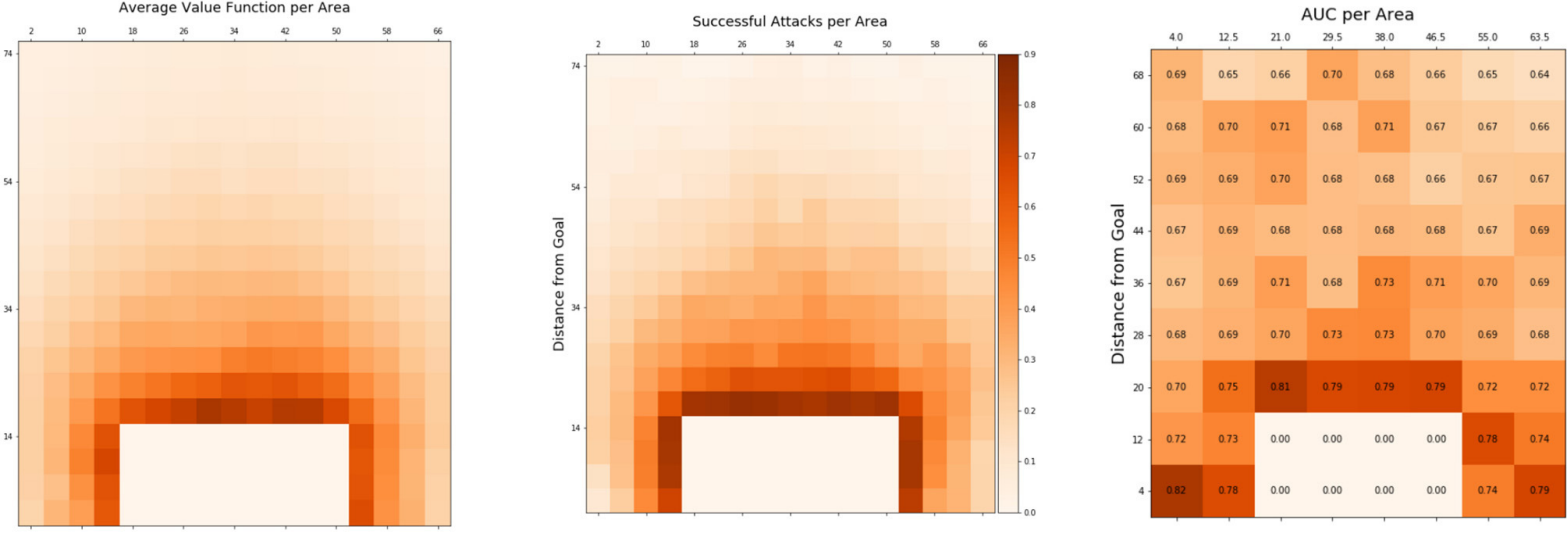
Value function (V) and observed success depending on ball position. Depicted are the distance from the goal line (y-axis) and distance from the sideline (x-axis). **(Left)** average V per ball position. **(Center)** true average success rate of attacks after the ball passes through an area. **(Right)** AUC values comparing V as a predictor for true success.

Trivially, the potential for a successful ball possession highly depends on the ball position: the closer to the goal of the opponent's, the higher the likelihood of success. This can be verified in Figure 2 (left) by visualizing the V depending on the position of the ball. The closer the ball to the goal is, the higher the V will be. This correlates highly with the true average success rate of attacks that went through certain areas on the pitch shown in Figure 2 (center). This observation is in line with other studies on dangerous in soccer, e.g., Link et al. (2016). On the other hand, the V is not very informative by itself, and we are more interested in whether high values correlate with higher success likelihoods on a per-attack basis. A sensible V should evaluate game situations based on the full configuration of player and ball positions and their recent past.

We test for both demands by relating the V to successful outcomes of ball possession phases by measuring the area under the ROC curve (AUC). To evaluate for predictability of success *independent of ball position*, we compute AUCs for small areas of the pitch individually. That way, ball position is roughly the same inside an area and differences in V stem only from ball and player positions and trajectories of the recent past. The results are shown in Figure 2 (right).

**Figure 3:**
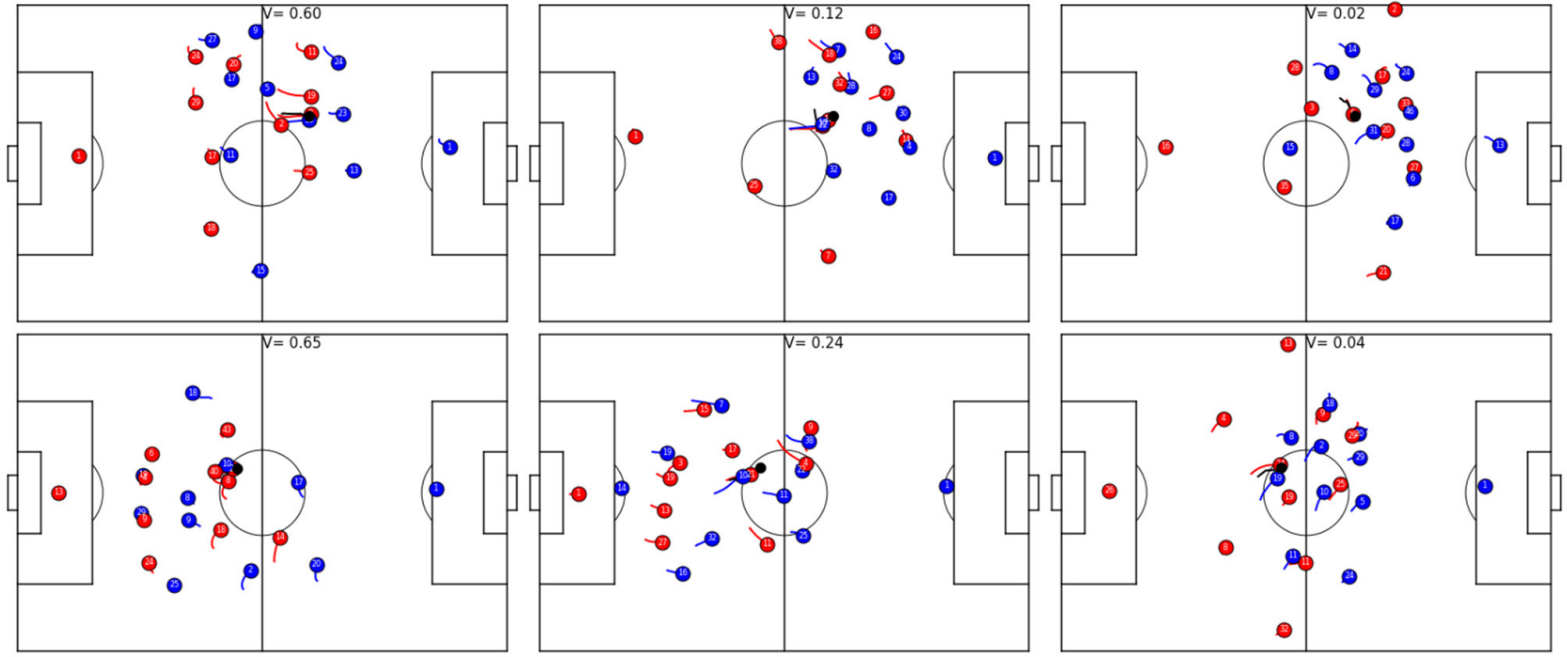
Exemplary game situations and corresponding V from different games. Red is always in ball possession and plays from left to right, the ball is shown in black and always at the same position in each row. **(Top row)**: red is starting a 5 vs. 4 counter-attacks (left, *V* = 0.6); the defending team is more organized and has more players behind the ball, but red player 27 is available for potential through ball (center, *V* = 0.12); the defending team is fully organized and the chance of a successful attack is slim (right, *V* = 0.02). **(Bottom row)**: the beginning of a counter-attack, where the left flank of team red potentially allows for a 3 vs. 1 situation (left, *V* = 0.65); potential counter-attack but both strikers are well covered and blue defender 11 is available to cover an attacker (center, *V* = 0.24); a fully organized defense (right, *V* = 0.04).

The figure displays higher AUC values for areas closer to the goal. An intuitive explanation is that, being so close to the dangerous zone, chance has less effect on the outcome of the ball possession phase as only a few more actions are required to successfully transfer the ball into that zone. Interestingly, the AUC never falls below 0.64, even well inside the own half of the attacking team (center-line is at 52.5 m). This may at first be considered a surprisingly good result, given that soccer is a highly unpredictable game where little things can change the outcome of a ball possession phase significantly. However, studying the results more carefully discloses that counterattacks, in general have high values accompanied by high success rates and often start deep inside the own half of the attacking team, which partly explains those good AUC values. Figure 3 shows examples of game situations and corresponding V including counterattacks that are rated with high values by the model.

**Figure 4:**
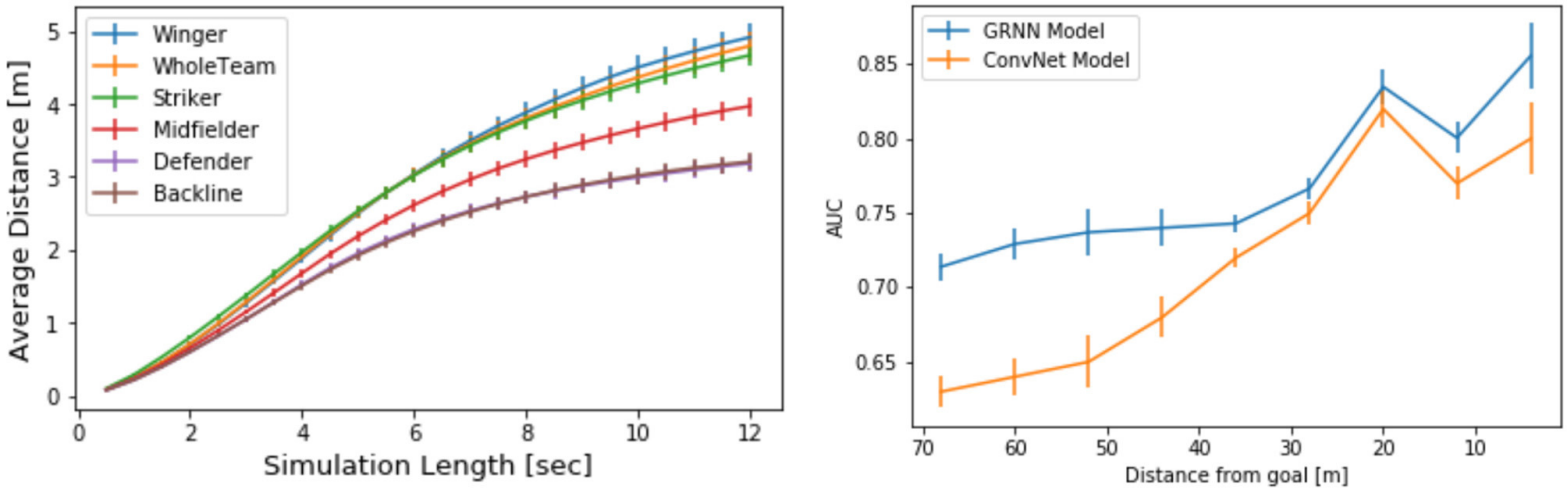
**(Left)** average distance between real and simulated player trajectories. **(Right)** comparison of AUC values of rating baseline (Dick and Brefeld, 2019) and this model along the length of the soccer pitch.

We also compare the approach to a baseline *ConvNet* originally presented in Dick and Brefeld (2019). The major difference between the two approaches is that the baseline learns a V using a convolutional neural network instead of the proposed GRNN. Figure 4 (right) clearly shows that this graph model significantly outperforms the baseline in terms of AUCs between true outcomes and the learned value functions. The results show that AUCs depend on the distance between the ball to the goal line (the *x*-coordinate), in contrast to the evaluation in Figure 2 (right) where the *y*-coordinate of the ball position was also considered. This evaluation is in line with the one presented in Dick and Brefeld (2019).

### 4.2. Movement Model

We now evaluate predictions of player trajectories of the model. The simulated movements should naturally be close to the real movements of a player in the same situations and depend on the movements of teammates, opponents, and the ball. Simulation is done by iteratively predicting the positions of players in 0.08 s (2 frames) and feeding those new positions back into the GRNN.

We empirically quantify deviations of simulations vs. ground truth by measuring the mean distance of *xy* positions of predicted trajectories and observed trajectories. Figure 4 (left) shows average deviations over future time intervals between 0.5 and 12 s.

Since we expect results to vary across different player roles, we provide different curves in the figure. The curves labeled defender, midfielder, winger, and *attacker* show the deviations of players of that category. Every curve shows averages and SE over several players but only one player is simulated at a time. This way, the differences in playing positions become obvious. Defenders need to be synchronized and their movement predictions are close to their real positions. Interestingly, simulating all defending players simultaneously, shown by the curve labeled backline, has the same deviation as the one for only a single defender. Forward players like wingers and strikers are the most difficult ones to predict, which is in line with the findings of Le et al. (2017a).

**Figure 5:**
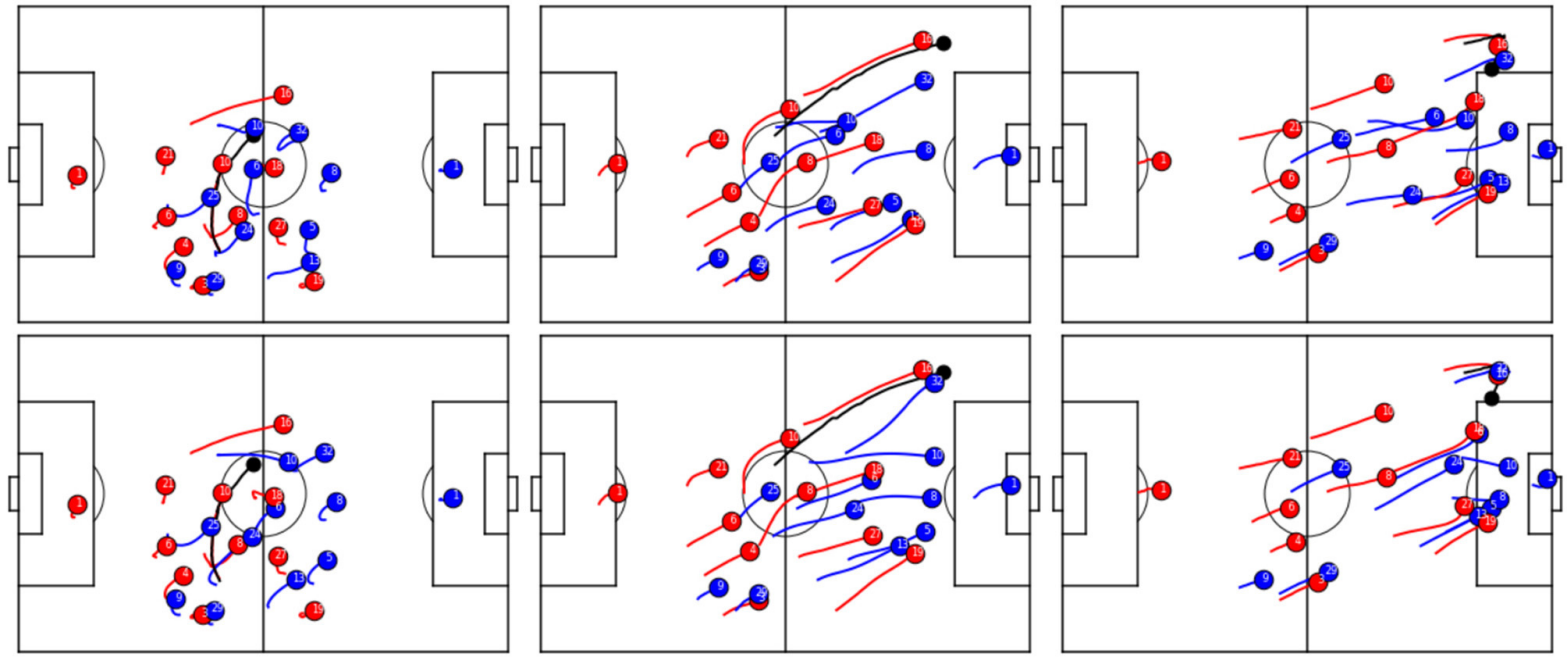
Real **(Top row)** vs. simulated **(Bottom row)** trajectories of the blue team.

The curve whole team shows the results for predicting trajectories of all players at a time. On average, a team has about three to five defenders, three midfielders, two wingers, and one to three strikers. The collective prediction ranges between that of individual wingers and strikers and works reasonably well. For all depicted curves, the average distance between predicted and real positions is roughly linear and less than a meter for 2 s, less than 2 m for 4 s, and so on. We compare the results to the best model of Yeh et al. (2019) in Table 1. The authors perform an extensive experimental evaluation, and the results suggest that the best model for predicting soccer trajectories is a graph network with Gaussian output; however, as Rudolph et al. (2020) show, using an MDN network can actually improve performance when used in conjunction with GRNNs on low-dimensional data like in this study. These results show that this is, indeed, true when predicting soccer player trajectories. For each of the six different settings, this model significantly outperforms the approach by Yeh et al. (2019), according to a paired *t*-test with *p* <0.01. Figure 5 shows an example for collective predictions over 9 s.

**Table 1:** Average ℓ_2_-distances and SE between observed and simulated players.

	**First 4 seconds**		
**Role**	**This Model**	**Yeh et al. (2019)**	**Cohen's d (95% CI)**
Winger	1.89 ± 0.07	2.09 ± 0.06	−2.33 (−3.47, −1.19)
WholeTeam	1.91 ± 0.07	2.16 ± 0.07	−2.44 (−3.35, −1.52)
Striker	1.97 ± 0.06	2.19 ± 0.07	−1.01 (−1.74, −0.27)
Midfielder	1.68 ± 0.06	1.93 ± 0.06	−1.36 (−2.13, −0.59)
Defender	1.53 ± 0.04	1.67 ± 0.04	−2.38 (−3.28, −1.47)
Backline	1.51 ± 0.04	1.70 ± 0.05	−3.28 (−4.34, −2.22)
	**First 8 seconds**		
**Role**	**This Model**	**Yeh et al. (****2019****)**	**Cohen's** **d** **(95% CI)**
Winger	3.89 ± 0.14	4.36 ± 0.13	−2.10 (−3.19, −1.00)
WholeTeam	3.81 ± 0.14	4.66 ± 0.16	−4.01 (−5.21, −2.80)
Striker	3.77 ± 0.11	4.18 ± 0.12	−0.92 (−1.64, −0.19)
Midfielder	3.25 ± 0.10	3.67 ± 0.11	−1.44 (−2.22, −0.66)
Defender	2.73 ± 0.10	3.23 ± 0.11	−2.19 (−3.07, −1.32)
Backline	2.73 ± 0.10	3.32 ± 0.11	−4.15 (−5.38, −2.92)

### 4.3. Quantifying Player Performance

Scouts, game analysts, and media experts are often interested in individual player behavior and try to measure each contribution of player to the outcome of ball possession phases and, ultimately, the game. On-ball actions are often easier to valuate, e.g., by measuring simple metrics, such as pass completion rates, turnovers, assists, and goals. But those metrics may not tell the whole story and decisive actions, such as an opening pass that leads to a good shooting chance three passes later may be overlooked.

Off-ball actions, such as, running into open space, creating space for teammates by moving away from certain areas of the pitch, reducing passing options by intelligent runs by defenders, or simply good man-marking, can be even harder to rate, even for professional analysts and coaches. While rating some of those actions may only be possible by the coaching staff because they strongly depend on team tactics and a specific task of the player in a given situation, we still hypothesize that rating a contributions of the player to the team based on the overall likelihood of success of the team, measured by the V, can introduce a new objective means in the assessment of performance of the player.

#### 4.3.1. On-Ball Actions

**Figure 6:**
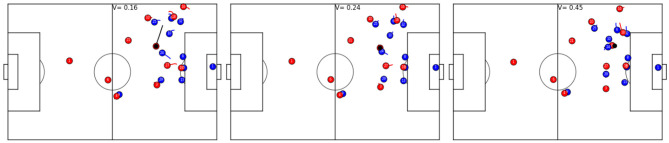
Temporal development of the V for a dribble. Red is attacking from left to right. Player 8 receives a pass from the left-wing; blue player 24 moves toward ball, *V* = 0.16 **(Left)**. Blue player 24 attacks the ball carrier, though from a non-optimal angle, yielding *V* = 0.24 **(Center)**. The ball-possessing player dribbles past the defender and the V jumps to *V* = 0.45 **(Right)**.

**Figure 7:**
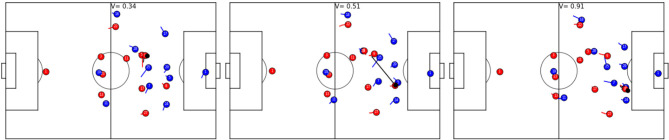
Temporal development of the V for a passing action. Red is attacking from left to right. Player 9 is about to pass the ball to player 8 **(Left)**. There is no immediate pressure on player 9 and the V is 0.34. Player 8 receives the ball under heavy pressure by blue player 5, which yields *V* = 0.51 **(Center)**. Receiver 8 was able to retain possession of the ball and is dribbling into the dangerous area, reflected by *V* = 0.91 **(Right)**.

On-ball actions have the most influence on the outcome of a ball possession phase. Passing a dangerous through-ball, dribbling past a defender, or simply finding an open teammate for a simple back-pass decide the development of a ball possession phase. This is mirrored in the V which is highly influenced by the ball position. As a consequence, on-ball actions are well captured by the temporal development of V values. Figure 6 shows an example of such a temporal development of the V during a successful dribble. The example shows that a successful dribble can change the V significantly, and these changes can be largely attributed to the corresponding actions (Besides general defender positioning and poor defensive behavior of the blue player 24). Figure 7 on the other hand, exemplifies the evolution of the V during a successful pass. Again, the change in value can be largely attributed to the ball action.

We, now, aggregate situative “dribble effectiveness” and “pass effectiveness” scores of all players in the data, who took part in at least two full games. For that matter, we define a “dribble” by a player as the interval between the reception of the ball until loses the possession of the ball, usually by passing to a teammate. We define a “good dribble” as one that increases the V by at least 0.06 between the start and the end of the dribble. Smaller values introduce noise into the ranking, higher values simply thin out the number of situations that are taken into account. Analogously, a “good pass” increases the V by at least 0.06 between the time of the pass and the reception.

Tables 2, 3 show the 10 best players according to their dribble and pass effectiveness, respectively. We like to stress the point that this dataset consists of only 54 games, and several players simply do not have enough games in the dataset to qualify for the ranking. Also, the model is trained to estimate whether a ball possession phase reaches the dangerous zone just outside the penalty box, so that many strikers may not appear on the lists, just because they typically operate closer to the goal. Hence, the listed players mainly operate as wingers or attacking midfielders, and the only exceptions being two strikers, a defensive midfielder, and a full back.

**Table 2:** Dribble Ratings.

	**#Good** ** Dribbles**	**#Dribbles**	**Minutes ** ** Played**	**Good Dribbles ** ** per Minute**
Kingsley Coman	66	416	845	0.078
T. Ito	43	274	687	0.063
A. Rebic	36	186	615	0.059
Jadon Sancho	11	104	207	0.053
A. Robben	45	388	848	0.053
F. Rib?ry	20	229	429	0.047
L. Rupp	18	156	389	0.046
A. Harit	39	340	847	0.046
N. Amiri	38	390	969	0.039
N. Schulz	46	495	1,190	0.039

*Good Dribbles” are defined in the text*.

**Table 3:** Pass Ratings.

	**#Good ** ** Passes**	**#Passes**	**Minutes ** ** Played**	**Good Passes ** ** per Minute**
Jadon Sancho	12	51	207	0.058
Thiago	39	413	718	0.054
L. Rupp	21	90	389	0.054
K. Demirbay	30	178	603	0.050
V. Grifo	15	93	346	0.043
L. Stindl	37	235	897	0.041
S. Rudy	31	296	759	0.041
C. Tolisso	30	349	807	0.037
Michael Cuisance	19	142	519	0.037
A. Robben	31	197	848	0.037

“*Good Passes” are defined in the text*.

The first thing to note is that FC Bayern Munich dominates the rankings, which may not come as a surprise. Kingsley Coman, Arjen Robben, Franck Ribery, Thiago, Corentin Tolisso, and Sebastian Rudy have played for Bayern Munich during that season and make up a good portion of both top tens. To get a better understanding of the rankings, we compare them to ratings from the leading German soccer magazine kicker^5^, who rates all players of the Bundesliga two times each year. Indeed, Kingsley Coman was rated the second-best winger during winter rating of the kicker's, and Frank Ribery took the same position in the summer rating, with Arjen Robben being third in both cases. Thiago was considered the fourth-best attacking midfielder and Corentin Tolisso in the sixth. Sebastian Rudy, however, was only rated as the 12th best holding midfielder but was still able to have good pass effectiveness according to this model. Jaden Sancho of Borussia Dortmund, who appears in both top ten lists, only had seven full games during that season as a 17-year-old, and was thus not eligible for a kicker rating, but is considered one of the best young wingers in the world at the moment. Ante Rebic was named the fourth best striker and Lars Stindl the eighth despite becoming a German international the previous year.

Amine Harit was voted third-best attacking midfielder in the summer with Karim Demirbay at 12th. Nico Schulz is the only defensive player in our rankings and was ranked best full-back in the summer. Four players could be considered surprise entries, Tatsuya Ito, Michael Cuisance, Vincenzo Grifo, and Lukas Rupp, who also appeared in both lists. Tatsuya Ito had his breakthrough season at Hamburger SV as a 20-year-old where he played his first nine professional games in the second half of the season. Despite only playing half of all possible games, he was voted tenth-best winger in the summer. Michael Cuisance was 18 during that season and the second-youngest player ever to play for Borussia Monchengladbach. He was transferred to Bayern Munich at the end of the following season. Vincenzo Grifo played mainly as a winger for Borussia Monchengladbach and debuted for the Italian national team after the season. Lukas Rupp only played 17 games with at least 45 min of playing time during that season and his grades rank him in midfield among all midfielders of the Bundesliga; however, his position as an attacking midfielder for TSG Hoffenheim, who finished the season as third in the table, still helped him being ranked well according to this model.

#### 4.3.2. Off-Ball Actions

Off-ball actions are naturally harder to evaluate objectively, though there are player actions that can be readily evaluated using the V such as a lost defensive dribble, that could be identified by a negative gain in the V during that action. Another example is a successful pass reception where the receiver could be attributed with the gain in V during the pass because movements of receiver (at least in part) led to him being available for the pass.

In analogy to the previous tables, Table 4 shows the top 10 pass receivers in this data. Not surprisingly, only forwards make up the list, with James Rodriguez and Thorgan Hazard playing, in general an attacking midfield role and Thomas Müller playing all attacking positions. According to kicker ratings, Robert Lewandowski was considered the best striker with runner-up being Andrej Kramaric, Alfred Finnbogason being fifth and Guido Burgstaller being 7th. Thomas Müller was considered the best winger in the summer, James Rodriguez best-attacking midfielder and Thorgan Hazard was considered the fourth best winger. Breel Embolo was only considered the 15th best striker by kicker as a 20-year-old. A special mention may go to Leon Bailey, who did not qualify for the list because he only played 164 min in this data, but would have been top of the table otherwise (0.062 good receptions per minute). He was considered the best winger in the winter.

**Table 4:** Pass Receiver Ratings.

	**#Good** ** Passes**	**#Passes**	**Minutes** ** Played**	**Good Passes** ** per Minute**
PlayerID				
Breel Embolo	28	86	484	0.058
R. Lewandowski	67	210	1,174	0.057
A. Kramaric	60	249	1076	0.056
James Rodr?guez	33	246	611	0.054
A. Finnbogason	13	35	245	0.053
G. Burgstaller	61	198	1,152	0.053
A. Rebic	31	112	615	0.050
T. M?ller	44	231	887	0.050
V. Grifo	17	101	346	0.049
Thorgan Hazard	51	281	1,128	0.045

“*Good Passes” are defined in the text*.

For many other circumstances, assigning a value to actions of thee player are less obvious and alternative approaches have to be used. One way to estimate a contribution of the player toward the potential for success of a game situation is by evaluating positioning of the player on the pitch, something which is often done during televised match analyses. The idea is to analyze how a decisive action, such as a forward pass, would have developed, or if it would have happened at all, if a certain player, often a defender, would have been positioned otherwise. We show that this model can be used to estimate quality of individual player movements and positioning and thereby quantify a influence of the player on the V.

The simplest way to use this model in that way would be to change the position of the player in the data artificially, i.e., change the *xy* coordinate of that player for the specific frame, and use the new V prediction of the model to estimate whether the new positioning would potentially affect the outcome of the ball possession phase. However, as the deep graph model employs recurrent GRU units, simply changing a position of the player could change the outcome of the prediction in undesired ways because the “simulated" trajectory would be physically impossible, e.g., by changing the position between two frames (0.04 s) by 2 m. Manually simulating trajectories of the player are possible but can be tedious.

Instead, we propose to evaluate off-ball actions of players by comparing their movements to movements of the average (Bundesliga) player that is naturally approximated by this model. As described above, this model is able to predict player trajectories realistically and can be used to simulate how a player could have acted instead. Comparing the temporal development of value functions between observed player trajectories and those where a trajectory of the player is replaced by the simulated one can be used to evaluate the (relative) quality of the observed actions.

**Figure 8:**
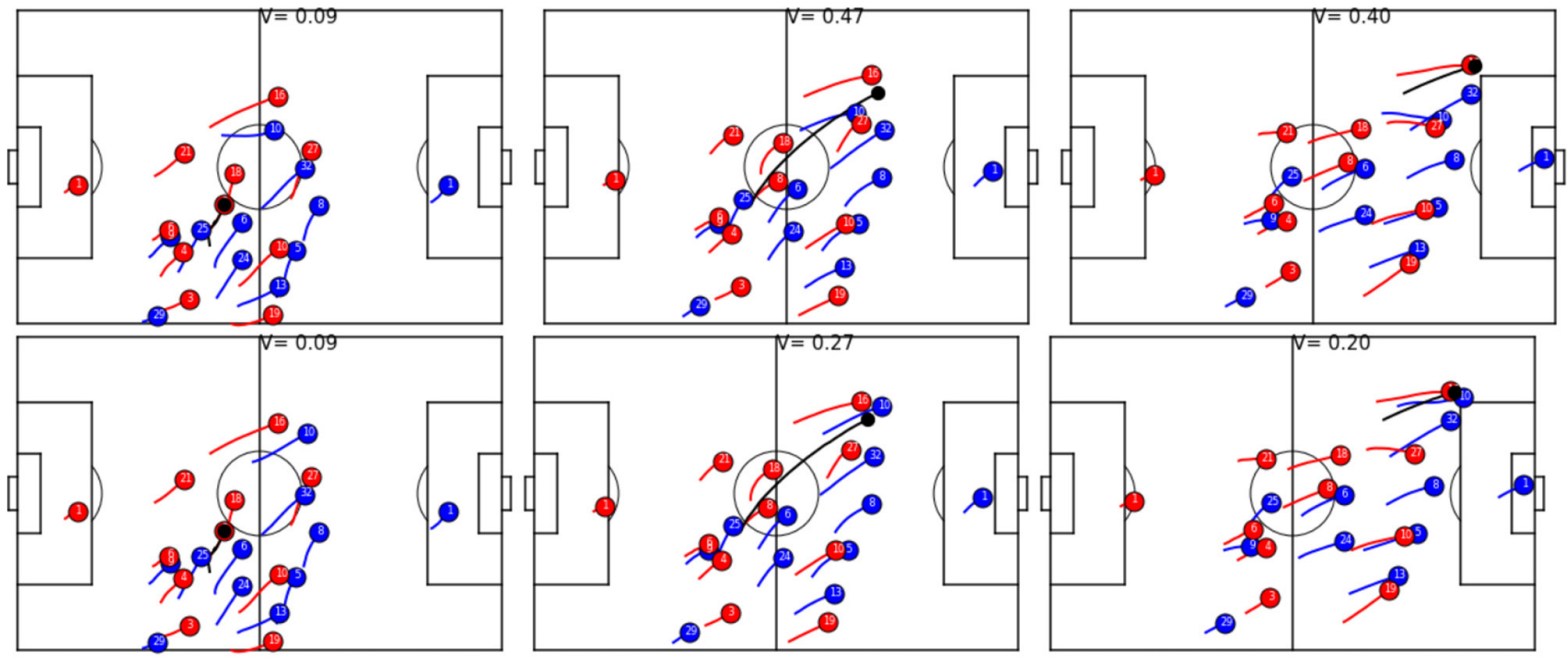
Temporal development of the V for observed trajectories **(Top)** and when one player (10, blue) is simulated **(Bottom)**.

Figure 8 shows an example of developing value functions of observed and simulated trajectories. In this article, we compare an observed trajectory of player 10 of the blue team to a simulated player and track the differences in V. The sequence starts after red gained ball possession (we omitted the first 2 s of the sequence in the plots for better readability). Immediately, both, the real player and the simulated player move back (left); however, the simulated player aims at taking a more defensive position and runs back quicker. The effect of this can be seen in the central figure, where the simulated player has a head start on the blue attacker 16 which reduces the V significantly for the simulation (27 instead of 47). The right figure shows that the observed player actually has no chance in putting pressure on the attacker and decides to cover red 27 instead. The simulated player, on the other hand, would have had a chance to get possession of the ball and stop the attack, leading to a lower V (20 instead of 40).

**Figure 9:**
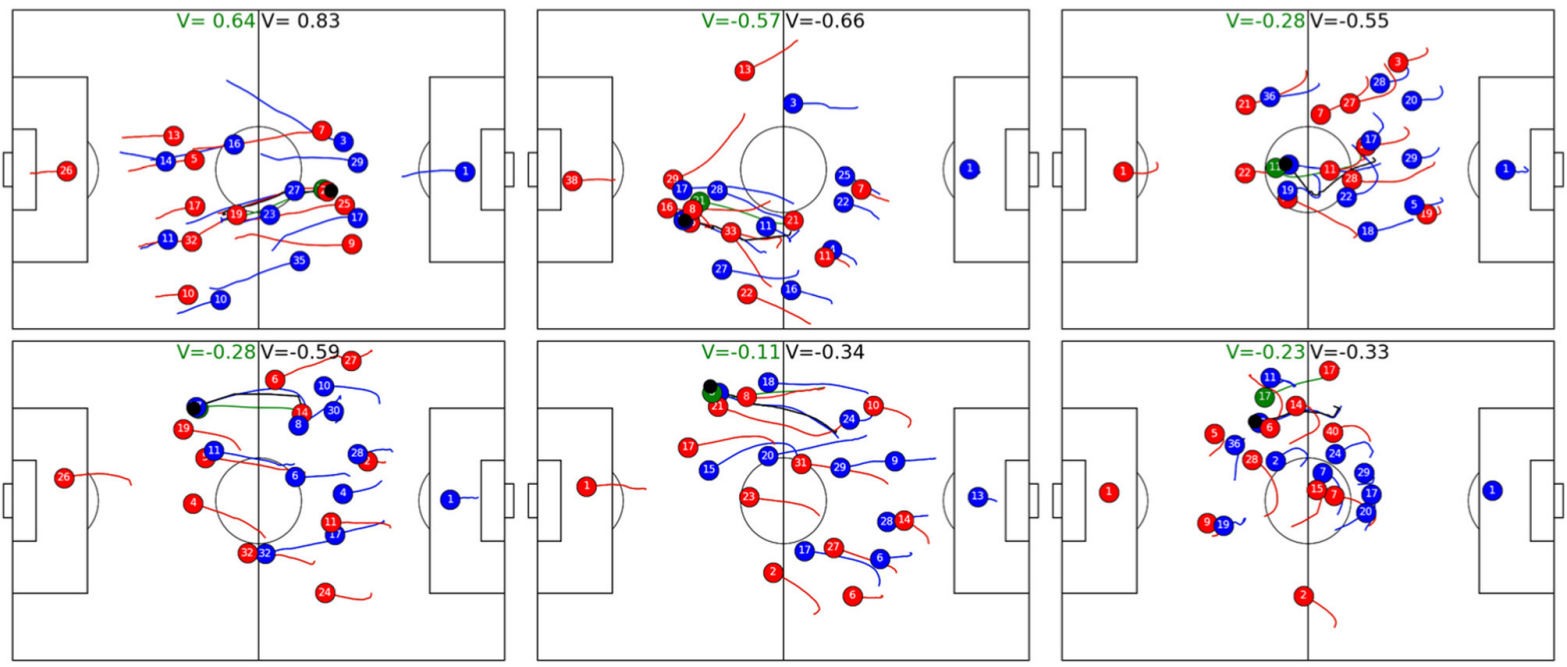
Simulated and observed trajectories of midfielders **(Top)** and wingers **(Bottom)** with the largest difference between observed and simulated V. The simulation starts 8 frames (0.32 s) after the opponent gaining possession of the ball and spans 4 s. Simulated players are depicted in green and V with simulated players are also labeled in green. In all cases, simulated players immediately tracked back after a loss of ball, whereas observed players either did not track back at all or reacted too late. The simulated player lost the ball in panels 1 (23, blue), 2 (21, red), 3 (11, red), and 4 (14, red). In panel 5, red player 21 lost the ball and 8 of red is simulated, whereas in panel 6, blue no. 12 (partly occluded) intercepted a pass and red no. 17 is simulated.

**Figure 10:**
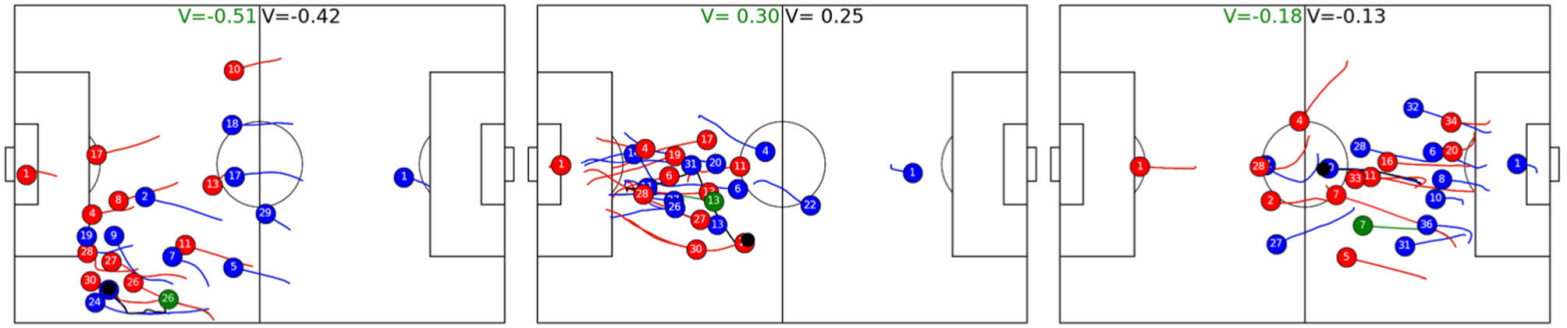
Simulated and observed trajectories of midfielders and wingers with largest negative difference between observed and simulated V. The simulation starts 8 frames (0.32 s) after the opponent gaining possession of the ball and spans 4 s. Simulated players are depicted in green and V with simulated players are also labeled in green. In all cases, the simulated player tracked back slower after the opponent gained ball possession, yielding worse value function from the perspective of the defending team. In panel 1, observed red 26 added additional pressure on the ball carrier, whereas the simulated player is too far away. Panel 2 shows an instance where blue 13 is again closer to the ball-possessing player and reduces options for the players. In the last panel, red 7 runs back in full speed to put pressure on the ball carrier, whereas the simulated player runs back slower, effectively having no impact on the developing ball possession phase.

Table 5 shows how much movement of a single player's from the defending team can change the predicted outcome of a ball possession phase. During a ball possession phase, we simulate player trajectories and compute the difference in absolute V values between the ball phase with the observed player vs. the possession with the simulated player. A positive difference denotes that the average absolute value including the observed player is larger than the one including the simulated player. The first thing to note is that average distances of V are very small (≈10^−4^), which again shows that simulated players behave very similar to observed ones; however, while during non-successful ball possessions—i.e., successful from the point of view of the defender, the average values of simulated players are more or less indistinguishable from observed one, simulations are on average a bit better during successful ball possession phases, unsuccessful from the point of view of the defender. In other words, during successful attack, players of the defend team sometimes behave worse than the average player and thereby increase the likelihood of the other team running a successful attack. Interestingly, according to the model, midfielders, wingers, and attackers a had stronger negative influence on the outcome than defenders. Figure 9 shows the reason. The figure shows the six instances where a midfielder or attacker behaved worst compared with the average player. In all six cases, the suboptimally behaving player does not track back immediately after the opposing team gets ball possession, whereas the simulated average player would run back at high speed. We like to note that accelerations and speeds are always well within realistic bounds, as e.g., defined in Peralta Alguacil et al. (2020) and learned implicitly by this model; however, Figure 10 shows that there are instances where the observed midfielders and wingers reacted faster than the average player, which leads to better V. The figure depicts the three instances where the observed player behaves the best relative to the simulated one, as measured by V difference.

**Table 5:** Average value function difference between simulated and observed trajectories categorized by approximate position.

	**Mean Difference in** ****V**** **(**×10^**−4**^**)**	
**Role**	**Non-successful possession**	**Successful possession**
Defense	–0.0	0.4
Midfield	0.1	1.0
Striker	0.2	0.8
Winger	0.2	1.0

*Only players of the defending team are considered*.

## 5. Applications

The previous section shows that these contributions accurately predict the movements of players as well as the potential for success of a game situation. We also indicate that there are various use-cases, where temporal changes in the V can be credited to a player or team performance; however, there are certainly many more applications where the use of this approach could be beneficial.

The previous section also indicates the potential for computation of novel KPIs, such as the conversion rate of dangerous situations into goals and/or shots, attributed to both teams and players. Other performance metrics could measure where most of potentially dangerous attacks of a team originate or in which areas a team is most dangerous. Those metrics are more general than common indicators that measure where successful (in the sense of a shot or goal) attacks originate or develop.

The V and derived performance indicators can also be used in retrieval systems to quickly find situations of interest. While trivial situations like corner and free kicks, shots, or passes are already annotated in the event data, complex events, such as a pressing situation or possibilities for counter-attacks are often tedious to identify and require human effort. Particularly for opponent analysis or scouting, this approach may come in handy since queries asking for the dangerous situations in a certain part of the pitch or good actions of a certain player can be processed automatically. As an example, potentially dangerous counter-attacks could be retrieved by querying for high V values inside a own half of a team. On the other hand, querying for situations where a team gains possession of the ball just inside their own half (and e.g., only 4 players of the opponent behind the ball) but low V could return sets of game situations that show how well a team is positioned against counter-attacks.

A limitation of this approach is clearly the data. Players are represented only by their coordinates on the pitch while their pose is ignored. Hence, it is not always clear whether players are standing or lying, walking forward or backward, and a player who looses the ball due to a tackle may be pushed to the ground (without being fouled) in the course of the action. In that case, the player is physically not able to track back immediately, a fact that is unknown to the model.

Due to the conceptual approach, this model provides the same solution for all players in the same situation. It is not personalized in the sense that it remembers players across games or takes history of a player's during a game into account. Thus, it is oblivious to a state of the player regarding mental and physical fatigue. On one hand, personalization would require access to additional data sources, such as for measurements of the mental or physical state of the player including load monitoring, self-perception of fatigue, stress, hours of sleep, muscle pain; however, the incorporation of such “orthogonal” data also opens up new possibilities, since the model could learn about individually preferred movements and physical and mental strength. Such additional information could easily be integrated into the model by either incorporating a player embedding layer for personalization or by adding additional physiological data into the input function ϕ_*I*_ (Equation 6).

## 6. Conclusion

We presented a data-driven model for predicting player trajectories and estimating the potential success of a game situation in soccer. The model employs a GRNN that effectively captures relations about all players and the ball and enabled us to make predictions based on the state of the game. Empirically, we observed that the model learned realistic movements of players and significantly outperformed a baseline approach and that learned V are a good predictor of the outcome of ball possession phases. We derived novel performance indicators by inspecting temporal developments of the V and showed that those correlated well with human expertise. In addition, by combining trajectory and success prediction, we were able to identify the sub-optimal behavior of players.

## Data Availability Statement

The data analyzed in this study is subject to the following licenses/restrictions: The data is owned by DFL and must not be shared with 3rd parties. Requests to access these datasets should be directed to www.dfl.de.

## Author Contributions

All authors listed have made a substantial, direct and intellectual contribution to the work, and approved it for publication.

## Conflict of Interest

The authors declare that the research was conducted in the absence of any commercial or financial relationships that could be construed as a potential conflict of interest.

## Footnotes

^1^e.g., https://www.bundesliga.com/en/bundesliga/stats/players.

^2^c.f. Linke et al. (2020) for an empirical validation of the data.

^3^https://www.dfl.de/en/innovation/how-is-the-official-match-data-collected/.

^4^The model was implemented using TensorFlow and learning of a model took about 5 h on an Nvidia V100 GPU.

^5^www.kicker.de.
